# Prevalence, comorbidities, and treatment patterns of Japanese patients with alopecia areata: A descriptive study using Japan medical data center claims database

**DOI:** 10.1111/1346-8138.16615

**Published:** 2022-11-02

**Authors:** Eduardo Campos‐Alberto, Tomohiro Hirose, Lynne Napatalung, Manabu Ohyama

**Affiliations:** ^1^ Medical Affairs, Pfizer Tokyo Japan; ^2^ Medical Affairs, Pfizer New York New York USA; ^3^ Icahn School of Medicine at Mount Sinai New York New York USA; ^4^ Department of Dermatology Kyorin University Faculty of Medicine Tokyo Japan

**Keywords:** alopecia areata, comorbidities, demography, prevalence, treatments

## Abstract

Real‐world data on alopecia areata (AA) demographics, comorbidities, and treatment patterns are sparse, not only in Japan but worldwide. This cross‐sectional study assessed the current prevalence of AA in Japan, including analysis of severe subsets, frequency of comorbidities, and unmet medical needs surrounding treatment. Patients registered in the Japan Medical Data Center claims database (January 2012 to December 2019) and diagnosed with AA were included. Prevalence was calculated yearly, with the most common comorbidities evaluated, and treatments described in the Japanese Dermatological Association AA management guidelines and approved in Japan were included in the analysis. In total, 61 899 patients were diagnosed with AA. Among them, 1497 were diagnosed with severe subtypes. AA prevalence in Japan has been gradually increasing (from 0.16% in 2012 to 0.27% in 2019). The most common comorbidities are allergic rhinitis, atopic dermatitis, and asthma. Depression and anxiety are frequent in these patients, as are autoimmune diseases, e.g., vitiligo, thyroid diseases, and rheumatoid arthritis. Intriguingly, the analysis found Down syndrome to be a comorbidity associated with severe AA in children. The principal treatments were topical corticosteroids, followed by carpronium chloride and cepharanthine. The use of systemic corticosteroids and antihistamines is increased in severe disease. The Japanese Dermatological Association guidelines do not support the use of oral corticosteroids in children; however, in the database, this has been prescribed in up to 2.5% and 9.8% of all pediatric and severe pediatric AA cases, respectively. Despite the limitations of using a claims database, the current study demonstrates that AA prevalence in Japan has gradually increased in recent years, with allergic diseases being the most common comorbidities. The data also imply that there is a need for effective and safe therapies, especially for severe and pediatric cases.

## 1 INTRODUCTION

Alopecia areata (AA) is an autoimmune and clinically heterogenous, nonscarring hair loss disease with an underlying immunoinflammatory pathogenesis.^1^, ^2^ It is considered to be a T‐cell–mediated, autoimmune hair disease.^3^, ^4^ AA affects both adults and children ranging from one or more discrete, well‐circumscribed round or oval patches of hair loss on the scalp or body to total hair loss. It can affect the entire scalp (alopecia totalis [AT]) or the entire scalp, face, and body (alopecia universalis [AU]). It may also present with unique variants such as alopecia ophiasis (AO) pattern, characterized by the loss of hair in a band‐like shape at the circumference of the head, or widespread alopecia (WA).^1^, ^2^, ^5^ In Japanese clinical practice, WA refers to a loss of hair of 25% or more on the scalp of any cause. Although WA includes severe cases, it is not a clinical classification of AA, and most of these cases are considered to be AA (>25% hair loss is defined as severe AA by Japanese Dermatological Association [JDA] guidelines).^6^ The onset of AA occurs relatively early in life, with the reported peak incidence of AA occuring before 20 years of age and about 60% of cases experience their first manifestation of hair loss during their late childhood/early adulthood.^7^, ^8^ Data from secondary and tertiary referral centers indicate that 34% to 50% of patients recover within 1 year; however, almost all patients experience more than one episode of the disease and 4.5% to 36.1% of patients with AA may progress to AT or AU, from which full recovery is unusual (<10%).^9^, ^10^, ^11^

A recent cross‐sectional survey in the United States reported the prevalence of AA to be ~0.21% overall and 0.09% for moderate to severe disease.^12^ In a nationwide, cross‐sectional, seasonal, multicenter study in Japan (2007–2008), the prevalence of AA among 67 448 patients with skin disorders was 2.45%.^13^ Despite this, there have been no epidemiological studies that specifically focus on AA in a Japanese population.

Treatment options for AA can have limited success; there is no cure, and no therapy has been shown to prevent disease relapse.^1^, ^2^ Treatments include topical, locally injected, or systemic corticosteroids; topical immunotherapy; topical minoxidil; topical irritants such as anthralin; and systemic immunosuppressants such as cyclosporine and methotrexate.^14^ In Japan, as well as around the world, the foundation of AA treatment is corticosteroids; however, their efficacy and that of other available treatments (e.g., carpronium chloride hydrate, cepharanthine, monoammonium glycyrrhizinate/glycine/DL‐methionine [glycyrrhizin], and herbal medicine)^6^, ^15^ is low in patients with severe disease. Success rates vary overall, depending on the extent and duration of disease.^2^ Psychosocial support and therapy are also an important part of disease management, as patients with AA can find their disease psychosocially burdensome, and it has been frequently associated with psychiatric disorders such as depression and anxiety.^2^

Worldwide, demographic studies on AA are limited. To understand clinical gaps and unmet medical needs related to treatment, as well as the epidemiology of AA in Japanese patients, we conducted a real‐world study using a national claims database.

## 2 METHODS

### 2.1 Study design, data, and patient population

This was a retrospective, cross‐sectional study using data from the Japan Medical Data Center (JMDC) database, which is one of the largest claims databases in Japan that includes detailed inpatient, outpatient, and pharmacy data.^16^ Study participants included patients of all ages who had at least one claim with a diagnosis of AA (*International Classification of Diseases, Tenth Revision, Clinical Modification*, diagnosis code L63, and severe AA cases including AT code L63.0, AU code L63.1, AO code L63.2, and WA code L63.8, covering from January 1, 2012 to December 31, 2019). Patients must have been enrolled in health insurance for at least 6 months before and after the first diagnosis of AA to evaluate concomitant diseases and enrolled for 12 to 24 months after AA diagnosis to assess treatment patterns.

### 2.2 Outcomes

The prevalence of AA and of pooled severe AA subtypes ([AT, AU, AO] or WA) was evaluated by age (0–11, 12–17, 18–39, 40–59, and ≥60 years) and sex. The index date was the date of first diagnosis of AA, or first diagnosis of severe AA subtype or WA. If there were two or more records for a patient in the same year, the age registered in the first consultation was used for the analysis.

The frequency of comorbidities according to age, sex, and severity, i.e., AA or pooled analysis of severe AA ([AT, AU, AO] or WA) was assessed, as was the frequency and percentage of one comorbidity or two or more different comorbidities during the study period. The comorbidities evaluated were hyperthyroidism, hypothyroidism, vitiligo, atopic dermatitis, psoriasis, rheumatoid arthritis, ulcerative colitis, Crohn disease, type 1 diabetes, type 2 diabetes, iron deficiency anemia, pernicious anemia, anxiety, depression, androgenetic alopecia, hypertension, Down syndrome, lupus, asthma, allergic rhinitis, vitamin D deficiency, and metabolic syndrome.

The frequency and percentage of treatments use according to age, sex, and severity (individual analysis of AA, AT, AU, AO, and WA), and pooled analysis of the severe subtypes including WA, were evaluated. The treatment pattern was evaluated for 1 and 2 years after the index date (the day of the first diagnosis of AA or AT or AU or AO or WA). In addition, the number and percentage of patients who received one or more AA treatment during the study period were analyzed. According to the JDA AA management guidelines,^6^ the medications recommended to treat patients with AA are corticosteroids (injectable [intralesional, intravenous], oral, topical), glycyrrhizin (oral), carpronium chloride (topical), cepharanthine (oral), antihistamine (oral), and herbal medicine (oral). Use of these were evaluated in the study in addition to use of intramuscular corticosteroids. With the exception of antihistamines and intramuscular corticosteroids, all of these medications have been approved by the Pharmaceutical and Medical Devices Agency (PMDA) for the treatment of Japanese patients with AA.

### 2.3 Statistical analysis

Descriptive statistics including mean, standard deviation (SD), median, ranges (minimum to maximum), and interquartile ranges were used to summarize the data. Confidence intervals were calculated at a confidence level of 95% using the Clopper‐Pearson method.

The first date of observation was defined as the earliest date on which a patient's medical data were recorded during the study period, with the final date of observation being the last date on which a patient's medical data were recorded during the study period. Prevalence rate (percentage) was measured as (number of cases [new and old]/total population) × 100. All statistical analyses were performed using SAS version 9.4 (SAS Institute Japan Ltd.) and Amazon EMR (5.29.0) (Amazon Web Services, Inc.) and HUEs (4.4.0) (GitHub, Inc.).

## 3 RESULTS

### 3.1 Patient characteristics

Among 10023 033 patients recorded in the JMDC database from 2012 to 2019, a total of 61 899 were diagnosed with AA and, of these, 1497 were diagnosed with severe AA subtypes ([AT, AU, AO] or WA). The mean age at the time of AA diagnosis was 35.8 years (SD, 16.3 years), and 57% of patients were female. Approximately 70% of patients were diagnosed by a dermatologist (Table 1).

**TABLE 1:** Characteristics of patients with a diagnosis of AA between 2012 and 2019, according to the JMDC Claims Database

Characteristic	
All patients	*N* = 61 899
Mean age, years (SD)	35.8 (16.3)
Median age, years	38
Age range, years	0–74
Male, *n* (%)	26 634 (43)
Female, *n* (%)	35 265 (57)
Severe subtypes	
Alopecia totalis	*n* = 29
Mean age, years (SD)	34.3 (15.3)
Male, *n* (%)	9 (31)
Female, *n* (%)	20 (69)
Alopecia universalis	*n* = 406
Mean age, years (SD)	37.3 (14.7)
Male, *n* (%)	174 (42.9)
Female, *n* (%)	232 (57.1)
Alopecia ophiasis	*n* = 19
Mean age, years (SD)	34.4 (16.2)
Male, *n* (%)	4 (21.1)
Female, *n* (%)	15 (78.9)
Widespread alopecia	*n* = 1043
Mean age, years (SD)	35.9 (15.3)
Male, *n* (%)	446 (42.8)
Female, *n* (%)	597 (57.2)
Diagnosed by a dermatologist	Approximately 70%
New AA diagnosis	50 547
Male, *n* (%)	21 301
Female, *n* (%)	29 246

Abbreviations: AA, alopecia areata; JMDC, Japan Medical Data Center; SD, standard deviation.

### 3.2 Prevalence

**FIGURE 1:**
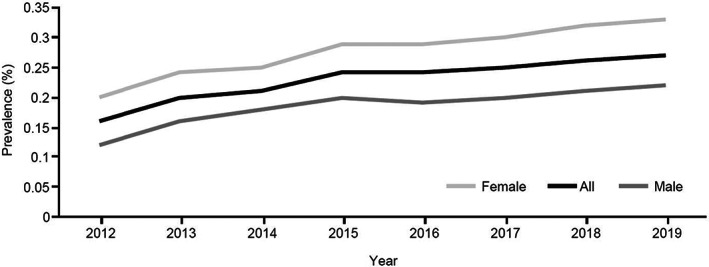
Alopecia areata (AA) yearly prevalence (percentage) trend from 2012 to 2019 according to sex. The prevalence rate (percentage) was measured as (number of cases [new and old]/total population) × 100. Data according to the Japan Medical Data Center Claims Database.

Prevalence was assessed yearly from 2012 to 2019, according to sex and age ranges. According to the JMDC database, AA prevalence has been gradually increasing in recent years, from 0.16% in 2012 to 0.27% in 2019 (Figure 1). Detailed prevalence data for AA and severe subtypes from 2017 to 2019 can be found in Table S1. The data show that AA is more prevalent in females and in adulthood; however, it is also frequently diagnosed during childhood.

Although the number of patients is small in comparison with the total AA population, the number of patients with severe subtypes ([AT, AU, AO] or WA) has gradually increased in recent years, although prevalence remains unchanged (Table S1).

### 3.3 Comorbidities

**FIGURE 2:**
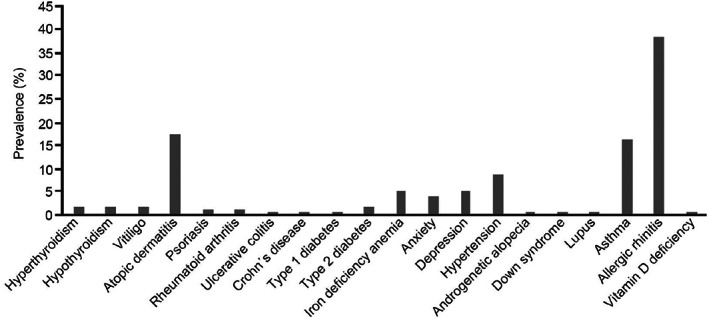
Prevalence of comorbidities in all patients with alopecia areata (AA) (*N* = 61 899) during the study period (2012–2019). Patients must have been enrolled in health insurance for at least 6 months before and after the first diagnosis of AA to evaluate comorbidities. Data according to the Japan Medical Data Center Claims Database.

**FIGURE 3:**
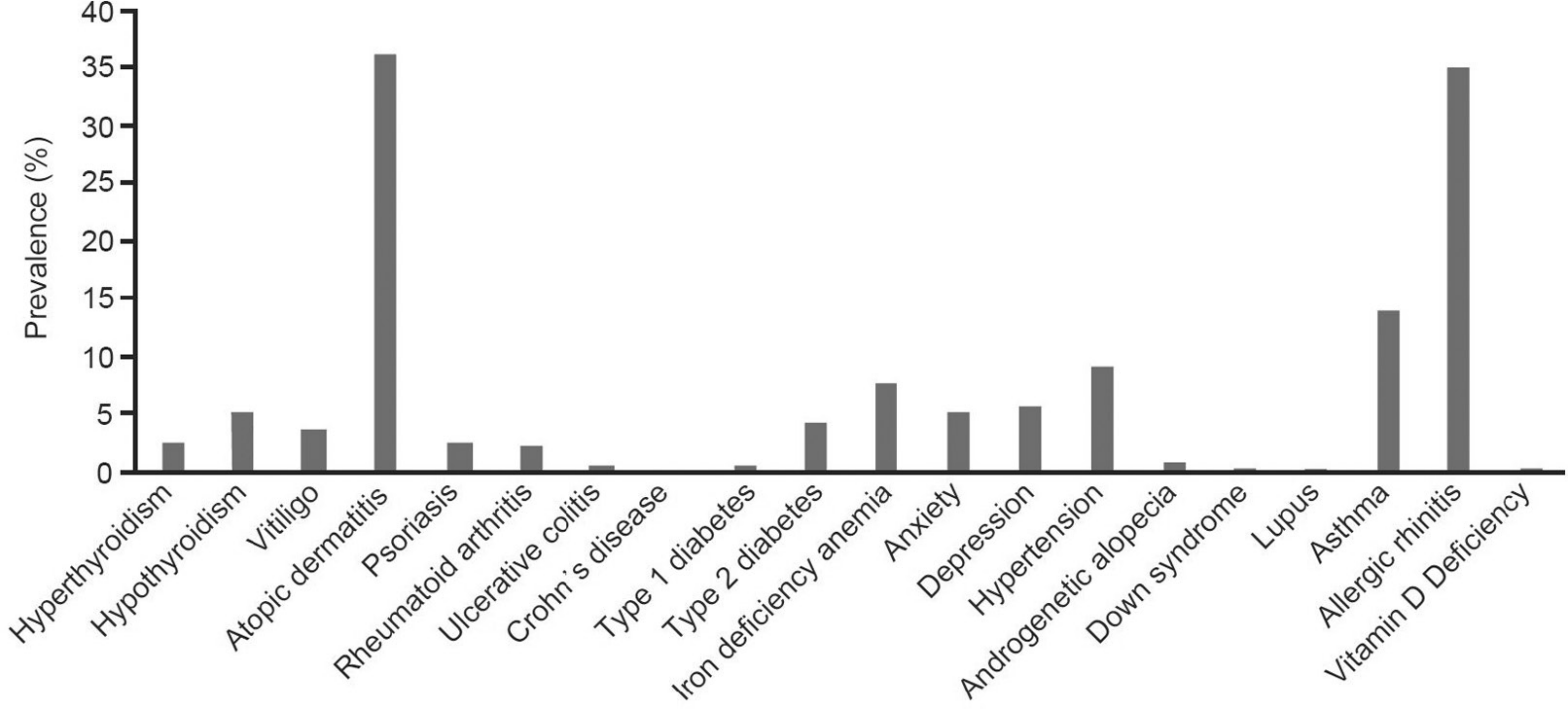
Prevalence of comorbidities in alopecia areata (AA) severe subtypes pooled (alopecia totalis [AT], alopecia universalis [AU], alopecia ophiasis [AO], or widespread alopecia [WA]) (*N* = 1497). Patients must have been enrolled in health insurance for at least 6 months before and after the first diagnosis of AT, AU, and AO subtypes, or WA, to evaluate comorbidities. Data according to the Japan Medical Data Center Claims Database.

The prevalence of comorbidities in AA and pooled severe subtypes ([AT, AU, AO] or WA) (Figures 2 and 3) was evaluated stratified by age range (Tables S2 and S3). The principal comorbidities in patients with AA were atopic diseases, such as allergic rhinitis (38.3%), atopic dermatitis (17.5%), and asthma (16.2%) (Figure 2), which are common in all ages but more frequent in children (Table S2). These atopic diseases were also the most common comorbidities in severe AA subtypes, with a high prevalence of atopic dermatitis (36.1%) and allergic rhinitis (35.1%) (Figure 3). Depression and anxiety were diagnosed often in patients with AA. Intriguingly, the analysis found that Down syndrome as a comorbidity in 0‐ to 11‐year‐olds increased from 0.6% in the AA group (Table S2) to 1.7% in those with severe AA (Table S3).

In general, in comparison with all AA cases, the frequency of all comorbidities was higher in the severe subtypes (Figures 2 and 3), including diseases such as hyperthyroidism (1.5% for all patients vs 2.5% for the severe group), hypothyroidism (1.9% vs 4.9%), vitiligo (1.6% vs 3.6%), psoriasis (1.3% vs 2.6%), rheumatoid arthritis (1.0% vs 2.3%), and iron deficiency anemia (4.9% vs 7.7%). A similar trend was found in patients diagnosed with type 1 diabetes (0.2% for all patients vs 0.6% for the severe group) and type 2 diabetes (1.9% vs 4.1%). There were more patients with at least two comorbidities in the severe AA subtype groups (39.1%) than in all AA cases (29.3%), for all age groups (Table S4).

### 3.4 Treatments

**FIGURE 4:**
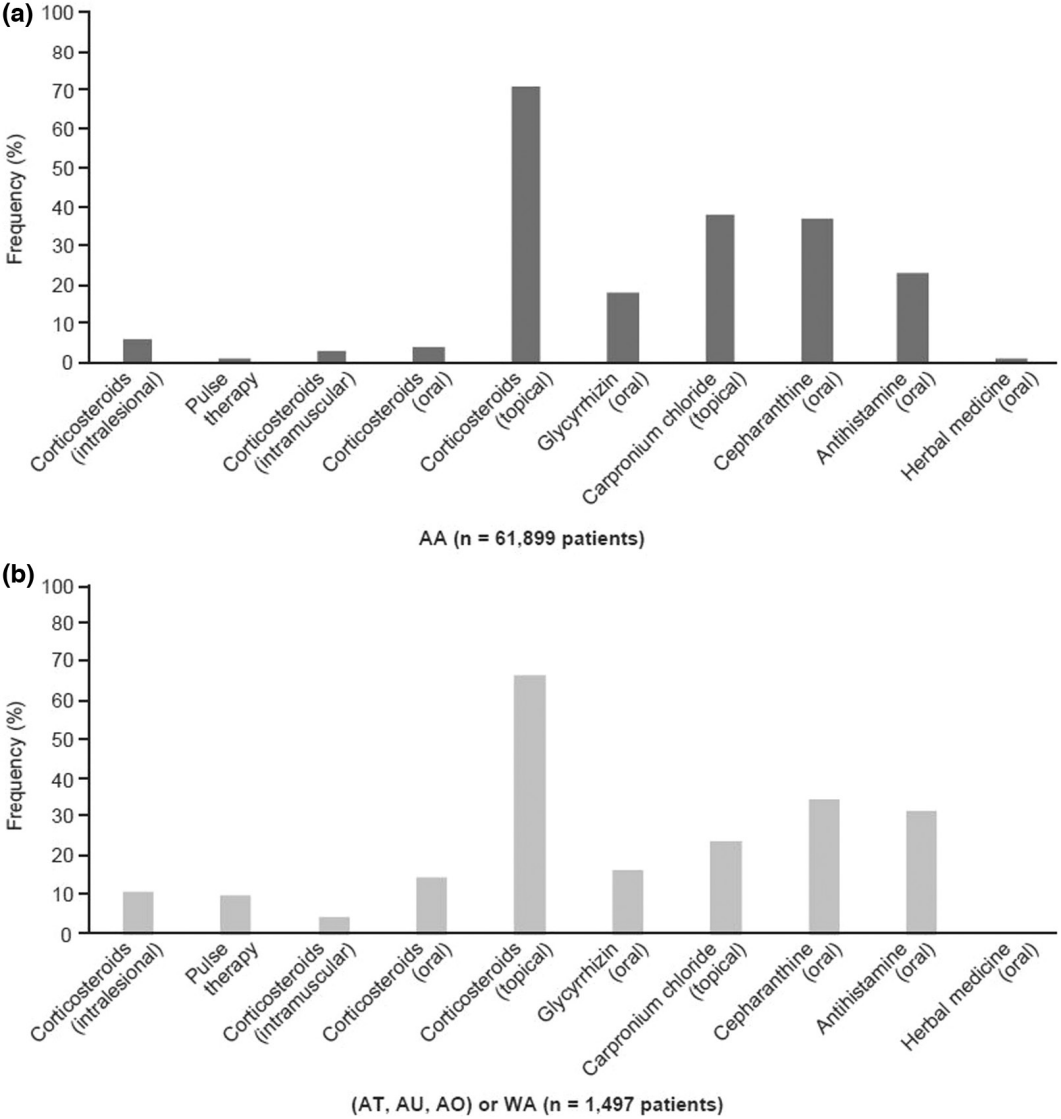
Treatment‐use frequency during 2 years after first diagnosis of (a) alopecia areata (AA) (*N* = 61 899) or (b) AA severe subtypes (alopecia totalis [AT], alopecia universalis [AU], and alopecia ophiasis [AO], or widespread alopecia [WA]) (*N* = 1497). Patients must have been enrolled in the health insurance for 12 months after AA diagnosis, to assess treatment patterns. Data according to the Japan Medical Data Center Claims Database.

Treatments used during the 2 years after diagnosis of AA or severe AA subtypes ([AT, AU, AO] or WA) (Figure 4) were assessed. The treatments most frequently prescribed to patients with AA were topical corticosteroids (70%), followed by carpronium chloride (37%) and cepharanthine (36%) (Figure 4a). The use of systemic corticosteroids (intralesional, intravenous corticosteroid pulse therapy [methylprednisolone, intravenous 500 mg/day for 3 days], intramuscular, oral) and antihistamines increased in patients with severe AA (Figure 4b).

**FIGURE 5:**
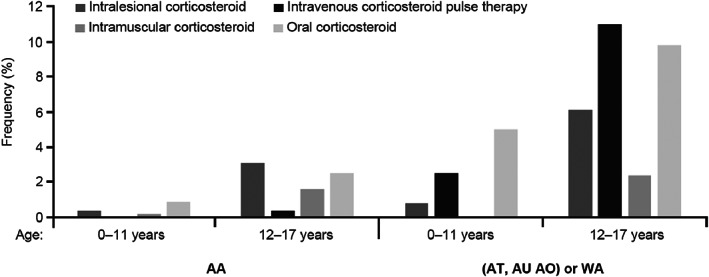
Use of systemic corticosteroids (intralesional, intravenous corticosteroid pulse therapy, intramuscular, oral) 2 years after alopecia areata (AA) diagnosis or severe AA subtypes (alopecia totalis [AT], alopecia universalis [AU], and alopecia ophiasis [AO], or widespread alopecia [WA]) diagnosis in the age groups 0–11 years and 12–17 years. Data according to the Japan Medical Data Center Claims Database.

In the analysis by age group according to AA or severe types ([AT, AU, AO] or WA) (Tables S5 and S6), after 2 years from diagnosis, the use of systemic corticosteroids increased in severe pediatric patients aged 0 to 11 years (Figure 5) from 0.9% oral corticosteroid use in those with AA to 5% in those with severe subtypes. This number increased from 2.5% to 9.8% in the children aged 12 to 17 years. Intravenous corticosteroid pulse therapy administration increased in the 12‐ to 17‐year age group from 0.4% for patients diagnosed with AA to 11.0% for those with severe subtypes; similarly, use of intramuscular corticosteroids increased from 1.6% in AA to 2.4% in severe subtypes in the 12‐ to 17‐year age group.

In general, intravenous corticosteroid pulse therapy was administered in inpatient settings (216 of 220 patients); in contrast, intramuscular corticosteroids were commonly administered in outpatient settings (1777 of 1778 patients).

During 2012 to 2019, topical corticosteroid utilization in patients with AA (Table S7) showed a slight increase. In the evaluation of severe subtypes (Table S8), increased prescription of intralesional and intravenous corticosteroid pulse therapy was observed in recent years. Although oral corticosteroid use has tended to decline gradually in recent years, it was prescribed to ~12% of patients with severe subtypes in 2019.

The frequency of patients with only one prescription was lower in the severe AA subtype groups versus all AA across all treatments (Table S9). The utilization of intravenous corticosteroid pulse therapy since start of treatment up to 2 years was also analyzed (Figure S1). A total of 220 patients received intravenous corticosteroid pulse therapy during the study period, and, among them, 85.4% were prescribed this therapy once only, 10.0% of patients twice, 3.2% three times, and 1.4% four or more times.

## 4 DISCUSSION

This is the first comprehensive study analyzing AA demographics, including yearly prevalence, comorbidities, and treatments commonly prescribed in Japanese patients with AA, covering an 8‐year period from the start of 2012 to the end of 2019.

The JMDC Claims Database provided a large sample of patients with AA from the general population who utilized medical services (*N* = 61 899); among these were severe cases of AA (*n* = 1497), defined as AT, AU, AO, or WA. Our results highlight that the prevalence of AA and its severe subtypes has gradually increased in recent years. In 2019, the prevalence of all AA cases was 0.27%, similar to prevalence figures reported in earlier studies from the United States. For example, Safavi^17^ described a prevalence of AA between 0.1% and 0.2% in the early 1970s. Recently, Benigno et al^12^ observed a clinician‐adjudicated AA prevalence of 0.21%. These results were different from those of Furue et al,^13^ who reported an AA prevalence of 2.45% in Japan; however, their prevalence study was of skin diseases in hospital settings, and this likely explains why they found a higher prevalence of AA. The prevalence of severe AA subtypes (including WA) in the present study was 0.01%, which is close to that obtained by Benigno et al^12^ in patients with AT/AU (0.04%), although their study was an online survey in a representative sample in the United States. In our study for the year 2019, AA was more prevalent in patients aged 40 to 59 years (0.34%), and it was more frequently diagnosed in females (0.33%) than males (0.22%); these results are consistent with the analysis obtained from the National Alopecia Areata Registry in a study on racial characteristics of AA in the United States,^18^ and with a report on sex differences in AA.^19^ Two population studies, however, cited no significant difference in the incidence of AA between males and females.^10^, ^20^

The most frequent comorbidities observed in this investigation were atopic diseases, especially allergic rhinitis and atopic dermatitis. These findings suggest a link between AA and atopy. Lee et al,^21^ in a systemic review and meta‐analysis of comorbidities in AA, also reported that patients with AA have higher odds of allergic diseases compared with controls without AA. Consistent with our results, Sung et al^22^ found that allergic rhinitis was the most common comorbidity of AA. Allergy disease history has been identified as a risk factor for increased AA susceptibility and a strong genetic link exists between AA and atopy (allergic rhinitis, atopic dermatitis, asthma) through the interleukin 13 and CLEC16A/KIAA0350 susceptibility loci, which have been identified in AA and other diseases such as psoriasis and arthritis.^23^

AA is an autoimmune disease that has been associated with other diseases, such as hyperthyroidism, hypothyroidism, vitiligo, psoriasis, and rheumatoid arthritis, as found in our study and other previous studies.^20^, ^24^ The increased risk of psoriasis in AA may be related to the upregulation of type 1 cytokines.^25^, ^26^ As for thyroid diseases, there is evidence of a higher rate of thyroid autoantibody positivity in patients with AA compared with controls without AA.^21^ Iron deficiency anemia, which was also found as a comorbid condition in our study, has previously been associated with a greater risk of autoimmune disease^27^ and has been linked as a prevalent condition in patients with AA; moreover, reduced ferritin levels have been reported in AA cases.^28^

Regarding hyperlipidemia, there have been conflicting results. A study in the US population^29^ listed hyperlipidemia as one of the most common comorbidities in AA. However, a report in Korea^21^ described that metabolic syndrome is associated with AA, but an association with hyperlipidemia (dyslipidemia) was not described. We investigated metabolic syndrome in the Japanese database and found only five male and two females were diagnosed with this condition, implying that the incidence of dyslipidemia could be distinct between the US and Japanese cohorts.

Other comorbid conditions in the present study included depression and anxiety. AA has been extensively associated with psychiatric disorders, which has been attributed to the chronic, relapsing nature of the disease and negative effect on patients' self‐esteem, considerably impacting on the quality of life.^2^ Affected individuals often describe AA as a life‐changing event, and the associated psychological distress may even lead to suicide, especially in patients with rapid‐onset alopecia.^30^ Awareness of these comorbidities is important for the management of AA or the referral of patients with AA to a psychologist or psychiatrist.

Pediatric patients with AA are more likely to have certain autoimmune and metabolic disorders than the general pediatric population.^31^ In our analysis, Down syndrome was a comorbidity, especially in children with severe AA (1.7%). This figure is higher than the estimated prevalence of Down syndrome in the general population (~0.1%).^32^, ^33^, ^34^ The association between AA and Down syndrome has been reported in the United States^35^ and England,^36^ and may be attributable to the relationship of organ‐specific autoimmune disease including antithyroid autoantibodies, which are often found in patients with Down syndrome,^36^ thus suggesting a possible link between autoimmunity and chromosomal disorders. In addition, an association between AA and an interferon‐inducible p78 protein gene (MX1), which maps to the distal part of the Down syndrome critical region, has been reported.^37^

The treatments analyzed in this study are approved by the PMDA for the treatment of Japanese patients with AA, the only exceptions being antihistamines and intramuscular corticosteroids. The most prescribed treatments for patients with AA were topical corticosteroids, carpronium chloride, and cepharanthine, with topical corticosteroids confirmed as the most frequently utilized treatment for AA at all ages. Corticosteroid efficacy in AA may be explained by the suppression of autoreactive immunocytes in AA.^38^, ^39^ Topical corticosteroids can be prescribed as a first‐line treatment (alone or in combination) to treat scalp, eyebrow, or beard AA.^40^ Topical corticosteroids may be beneficial in managing mild disease,^29^ and likely provide some benefit for patients with AA of limited disease extent.^41^ However, this treatment may be insufficiently effective in treating severe AA subtypes.^42^

Systemic corticosteroids (oral, intralesional, intramuscular, and intravenous corticosteroid pulse therapy) were also frequently prescribed, and the rate of use increased in patients with severe AA, including the pediatric population. Oral corticosteroids have demonstrated efficacy in stimulating hair regrowth in AA cases^41^; however, side effects include suppression of the pituitary–adrenal axis, effects on bone growth, osteoporosis, cataracts, immunosuppression, obesity, dysmenorrhea, acne, and worsening of hypertension and diabetes.^14^, ^43^ Hence, the use of oral corticosteroids must be carefully monitored and compliant with the JDA guidelines, which recommend using oral corticosteroids for a short period for rapidly progressive adult AA cases with hair loss affecting more than 25% of the scalp, with use in pediatric populations not recommended.^6^ Intralesional steroids are considered the standard of care for patchy AA of limited extent,^39^, ^41^ and although local injection of triamcinolone acetonide is relatively safe, it can cause adverse events such as skin atrophy, pain, and telangiectasia, and patients treated for long periods may be at higher risk of osteoporosis.^44^

According to the JDA guidelines, adults with active hair loss affecting more than 25% of total scalp, and with a duration of less than 6 months from onset, may be treated with intravenous corticosteroid pulse therapy, principally consisting of an inpatient single course of intravenous methylprednisolone (500 mg/day for 3 consecutive days).^45^ For patients with longer disease duration, or AT and AU phenotypes, poor outcomes with this therapy are reported.^39^, ^46^ Our results show that 220 patients utilized intravenous corticosteroid pulse therapy in a period of 2 years; among them, 85.5% received this treatment only once, as recommended,^45^ but 14.5% received it at least twice in this period, suggesting a need for more effective treatments. As previously mentioned, intravenous corticosteroid pulse therapy is commonly administered in specialized inpatient settings so the patient can be carefully monitored; however, the database listed five cases where such treatment was provided in the outpatient setting; this highlights the need to alert physicians to its potential adverse events, to secure medical safety.

The scarcity of effective medicines to treat AA may result in the use of treatments that are not supported by the JDA guidelines, such as intramuscular corticosteroids; although these were used mostly in outpatient adults in the current study, they were also prescribed in children. Chronic use of systemic corticosteroids, even at low doses, is associated with significant adverse consequences,^47^ and their use in children should be avoided.^6^

Other treatments that were found to be regularly used in Japan for treating patients with AA are carpronium chloride hydrate, cepharanthine, and glycyrrhizin. Hyperactivity of the sympathetic nerve may contract blood vessels around hair follicles, with consequent hair loss. Therefore, carpronium chloride, as a parasympathetic nerve stimulant,^15^ may increase blood circulation around hair follicles. Cepharanthine and its structural analogs have the potential to restore hair growth by promoting the proliferation of human dermal papilla cells and increasing their expression of vascular endothelial growth factor.^48^ It has been reported in vitro that glycyrrhizin acid, the active ingredient in glycyrrhizin tablets, is hydrolyzed by β‐D‐glucuronidase and metabolized to glycyrrhetinic acid.^49^ Since glycyrrhetinic acid has an inhibitory effect on 11β‐HSD2, the enzyme that metabolizes inactive cortisone to cortisol, it is potentially plausible that the inflammatory effects of cortisol in the body are indirectly affected by glycyrrhetinic acid, resulting in improvements in AA.^49^ Although these medicines have been approved for use in AA in Japan, putatively based on the rationales mentioned earlier, they are not commonly used for AA outside Japan and their grades of evidence are lower than for corticosteroids.^6^

Antihistamines are not approved for use in AA. However, they are preferentially used in patients with AA in Japan.^50^, ^51^ Histamine is thought to facilitate crosstalk with CD8 T cells, contributing to the collapse of follicular immune privilege observed in AA.^52^, ^53^ Antihistamines could downmodulate T‐cell chemotaxis toward CXCL10 by reducing chemokine receptor 3 expression, F‐actin polymerization, and calcium influx in patients with AA.^52^, ^54^ However, to demonstrate any proven benefit for antihistamine use in AA, more data are needed.

The present study benefits from a large population assessed over a long period, with most patients being diagnosed and treated by dermatologists. Limitations include the use of a claims database registry to analyze treatment patterns, where only medicines covered by health insurance in Japan can be evaluated. Hence, it was not possible to assess contact immunotherapy using squaric acid dibutylester and diphenylcyclopropenone, which is recommended by the JDA guidelines for moderate to severe AA cases in all age groups^6^, ^44^ with a report of a complete hair regrowth rate of 32.3%^44^; it would therefore be valuable to analyze this treatment using alternative study methodology. Another limitation is that AT, AU, and AO terms used within the claims database do not distinguish other hair diseases manifesting with similar phenotypes, e.g., ectodermal dysplasia for AU or frontal fibrosing alopecia for AO. However, the number of diseases that present similar clinical features to AU, AT, and AO is thought to be low. Moreover, telogen effluvium, male pattern hair loss, and female pattern hair loss are other conditions that may often be misdiagnosed and treated as AA. Patients with AA are sometimes misdiagnosed as having male pattern baldness and treated without insurance.

The JMDC Claims Database mostly includes health insurance data from people who are employed; thus, there are limited data registered for patients 60 years and older. However, a previous prevalence analysis of AA in Japanese patients who visited clinics or hospitals^11^ reported similar results to the current study for those 60 years and older. A future study assessing the impact of COVID‐19 from year 2020 on AA prevalence is warranted.

In conclusion, this large, comprehensive analysis reports that the prevalence of AA in Japan has been gradually increasing in recent years. There is also a high rate of comorbidities in patients with AA, principally atopic diseases, and the data highlight the need for more effective and safe therapies, especially for severe and pediatric cases.

## CONFLICT OF INTEREST

E. Campos‐Alberto, T. Hirose, and L. Napatalung are employees and shareholders of Pfizer Inc. M. Ohyama reports advisory fees from Pfizer Japan Inc, Eli Lilly Japan KK, Janssen Pharmaceutical KK, Taisho Pharmaceutical Co., and Rohto Pharmaceutical Co.; lecture fees from Eli Lilly Japan KK; research grants not related to this study from Maruho Co., Sun Pharma Japan Ltd, and Shiseido Co.

## Supporting information


Tables S1‐S9
