# Supplementary material for: Prevalence, comorbidities, and treatment patterns of Japanese patients with alopecia areata: A descriptive study using Japan medical data center claims database
Source: J Dermatol. 2022 Nov 2;50(1):37–45. doi: 10.1111/1346-8138.16615 (PMC10092019; doi:10.1111/1346-8138.16615)

**SUPPORTING INFORMATION**

**TABLE S1** Prevalence of AA and severe types pooled ([AT, AU, AO] or WA) according to age and sex (2017–2019)

| **Parameter** | **AA** | |  | **Pooled [AT, AU, AO] or WA** | |
| --- | --- | --- | --- | --- | --- |
|  | **n** | **Prevalence rate, % (95% CI)** |  | **n** | **Prevalence rate, %**  **(95% CI)** |
| **Jan to Dec 2017** |  |  |  |  |  |
| All | 15,561 | 0.25 (0.24, 0.25) |  | 456 | 0.01 (0.01, 0.01) |
| Male | 6752 | 0.20 (0.19, 0.20) |  | 195 | 0.01 (0.00, 0.01) |
| Female | 8809 | 0.30 (0.30, 0.31) |  | 261 | 0.01 (0.01, 0.01) |
| 0–11 years | 1490 | 0.15 (0.14, 0.16) |  | 35 | 0.00 (0.00, 0.00) |
| 12–17 years | 1060 | 0.22 (0.20, 0.23) |  | 31 | 0.01 (0.00, 0.01) |
| 18–39 years | 5093 | 0.24 (0.23, 0.24) |  | 166 | 0.01 (0.01, 0.01) |
| 40–59 years | 6843 | 0.31 (0.30, 0.32) |  | 203 | 0.01 (0.01, 0.01) |
| ≥60 years | 1075 | 0.23 (0.21, 0.24) |  | 21 | 0.00 (0.00, 0.01) |
| **Jan to Dec 2018** |  |  |  |  |  |
| All | 19,412 | 0.26 (0.26, 0.27) |  | 596 | 0.01 (0.01, 0.01) |
| Male | 8390 | 0.21 (0.21, 0.22) |  | 239 | 0.01 (0.01, 0.01) |
| Female | 11022 | 0.32 (0.32, 0.33) |  | 357 | 0.01 (0.01, 0.01) |
| 0–11 years | 1848 | 0.16 (0.15, 0.17) |  | 43 | 0.00 (0.00, 0.01) |
| 12–17 years | 1205 | 0.22 (0.21, 0.23) |  | 35 | 0.01 (0.00, 0.01) |
| 18–39 years | 6587 | 0.26 (0.25, 0.26) |  | 219 | 0.01 (0.01, 0.01) |
| 40–59 years | 8471 | 0.33 (0.32, 0.34) |  | 266 | 0.01 (0.01, 0.01) |
| ≥60 years | 1301 | 0.24 (0.23, 0.26) |  | 33 | 0.01 (0.00, 0.01) |
| **Jan to Dec 2019** |  |  |  |  |  |
| All | 20,405 | 0.27 (0.27, 0.27) |  | 672 | 0.01 (0.01, 0.01) |
| Male | 8793 | 0.22 (0.21, 0.22) |  | 278 | 0.01 (0.01, 0.01) |
| Female | 11,612 | 0.33 (0.32, 0.33) |  | 394 | 0.01 (0.01, 0.01) |
| 0–11 years | 1847 | 0.16 (0.15, 0.17) |  | 42 | 0.00 (0.00, 0.00) |
| 12–17 years | 1283 | 0.23 (0.22, 0.24) |  | 33 | 0.01 (0.00, 0.01) |
| 18–39 years | 6804 | 0.26 (0.25, 0.27) |  | 248 | 0.01 (0.01, 0.01) |
| 40–59 years | 9082 | 0.34 (0.34, 0.35) |  | 310 | 0.01 (0.01, 0.01) |
| ≥60 years | 1389 | 0.25 (0.23, 0.26) |  | 39 | 0.01 (0.00, 0.01) |

Formula for calculating the prevalence rate: (n/JMDC(n)) * 100.

95% CI was calculated using the Clopper-Pearson method.

Abbreviations: AA, alopecia areata; AO, alopecia ophiasis; AT, alopecia totalis; AU, alopecia universalis; CI, confidence interval; WA, widespread alopecia.

**TABLE S2** Demographics and comorbidities of patients with AA, stratified by age (N = 61,899)

|  | **Age group** | | | | |
| --- | --- | --- | --- | --- | --- |
| **Characteristic** | **0–11 y, n = 6657**  **(10.8% of patients)** | **12–17 y, n = 4199**  **(6.8% of patients)** | **18–39 y, n = 22,600**  **(36.5% of patients)** | **40–59 y, n = 24,757**  **(40% of patients)** | **≥60 y, n = 3686**  **(6% of patients)** |
| Male, n (%) | 2648 (39.8) | 1938 (46.2) | 10,436 (46.2) | 10,058 (40.6) | 1554 (42.2) |
| Female, n (%) | 4009 (60.2) | 2261 (53.8) | 12,164 (53.8) | 14,699 (59.4) | 2132 (57.8) |
| **Comorbidities during 6-month baseline period, %** | | | | | |
| Hyperthyroidism | 0.3 | 0.8 | 1.4 | 2.8 | 1.8 |
| Hypothyroidism | 0.9 | 1.4 | 1.5 | 3.4 | 3.9 |
| Vitiligo | 1.6 | 1.7 | 1.3 | 1.9 | 2.0 |
| Atopic dermatitis | 29.0 | 23.3 | 18.9 | 14.0 | 9.0 |
| Psoriasis | 0.5 | 0.7 | 1.0 | 1.7 | 2.6 |
| Rheumatoid arthritis | 0.1 | 0.1 | 0.6 | 2.3 | 2.8 |
| Ulcerative colitis | 0.0 | 0.1 | 0.3 | 0.3 | 0.1 |
| Crohn's disease | 0.0 | 0.1 | 0.1 | 0.1 | 0.0 |
| Type 1 diabetes | 0.1 | 0.1 | 0.1 | 0.2 | 0.2 |
| Type 2 diabetes | 0.1 | 0.3 | 0.6 | 2.4 | 8.2 |
| Iron deficiency anemia | 1.5 | 2.6 | 4.3 | 10.3 | 4.1 |
| Pernicious anemia | 0.0 | 0.0 | 0.0 | 0.1 | 0.1 |
| Anxiety | 0.6 | 1.9 | 3.6 | 5.9 | 6.8 |
| Depression | 0.3 | 1.6 | 5.1 | 6.7 | 5.7 |
| Androgenetic alopecia | 0.1 | 0.2 | 0.3 | 0.1 | 0.4 |
| Hypertension | 0.3 | 0.6 | 1.9 | 11.3 | 38.5 |
| Down syndrome | 0.6 | 0.3 | 0.1 | 0.0 | 0.0 |
| Lupus | 0.0 | 0.1 | 0.1 | 0.1 | 0.1 |
| Asthma | 45.0 | 18.5 | 11.2 | 14.3 | 14.9 |
| Allergic rhinitis | 62.8 | 47.9 | 33.3 | 38.1 | 36.2 |
| Vitamin D deficiency | 0.2 | 0.1 | 0.1 | 0.0 | 0.0 |
| Metabolic syndrome | 0.0 | 0.0 | 0.0 | 0.0 | 0.0 |

Abbreviations: AA, alopecia areata; y, years.

**TABLE S3** Demographics and comorbidities of patients with AA severe types pooled ([AT, AU, AO] or WA) stratified by age (N = 1497 patients)

|  | **Age group** | | | | |
| --- | --- | --- | --- | --- | --- |
| **Characteristic** | **0–11 y, n = 119**  **(8% of patients)** | **12–17 y, n = 82**  **(5.5% of patients)** | **18–39 y, n = 602**  **(40.2% of patients)** | **40–59 y, n = 617**  **(41.2% of patients)** | **≥60 y, n = 77**  **(5.1% of patients)** |
| Male, n (%) | 57 (47.9) | 38 (46.3) | 256 (42.5) | 244 (39.5) | 32 (41.6) |
| Female, n (%) | 62 (52.1) | 44 (53.7) | 346 (57.5) | 373 (60.5) | 45 (58.4) |
| **Comorbidities during 6-month baseline period, %** | | | | | |
| Hyperthyroidism | 1.7 | 1.2 | 3.2 | 2.4 | 1.3 |
| Hypothyroidism | 6.7 | 2.4 | 4.2 | 5.8 | 3.9 |
| Vitiligo | 5.0 | 2.4 | 3.8 | 3.4 | 2.6 |
| Atopic dermatitis | 52.9 | 40.2 | 39.5 | 31.0 | 20.8 |
| Psoriasis | 3.4 | 2.4 | 2.2 | 2.8 | 3.9 |
| Rheumatoid arthritis | 1.7 | 0.0 | 2.0 | 2.4 | 6.5 |
| Ulcerative colitis | 0.0 | 0.0 | 0.5 | 1.0 | 0.0 |
| Crohn's disease | 0.0 | 0.0 | 0.0 | 0.0 | 0.0 |
| Type 1 diabetes | 0.0 | 0.0 | 0.5 | 0.8 | 1.3 |
| Type 2 diabetes | 0.8 | 1.2 | 2.5 | 6.2 | 7.8 |
| Iron deficiency anemia | 2.5 | 2.4 | 8.5 | 8.9 | 5.2 |
| Pernicious anemia | 0.0 | 0.0 | 0.0 | 0.0 | 0.0 |
| Anxiety | 1.7 | 3.7 | 5.0 | 5.2 | 9.1 |
| Depression | 0.0 | 1.2 | 6.5 | 6.8 | 6.5 |
| Androgenetic alopecia | 0.8 | 0.0 | 0.8 | 0.6 | 0.0 |
| Hypertension | 0.0 | 0.0 | 2.8 | 13.6 | 45.5 |
| Down syndrome | 1.7 | 1.2 | 0.0 | 0.0 | 0.0 |
| Lupus | 0.0 | 0.0 | 0.5 | 0.2 | 0.0 |
| Asthma | 39.5 | 9.8 | 12.0 | 11.8 | 10.4 |
| Allergic rhinitis | 56.3 | 46.3 | 32.1 | 32.9 | 31.2 |
| Vitamin D deficiency | 0.8 | 0.0 | 0.0 | 0.0 | 0.0 |
| Metabolic syndrome | 0.0 | 0.0 | 0.0 | 0.0 | 0.0 |

Abbreviations: AA, alopecia areata; AO, alopecia ophiasis; AT, alopecia totalis; AU, alopecia universalis; WA, widespread alopecia; y, years.

**TABLE S4** Frequency of one and two or more comorbidities for AA and severe AA types pooled
([AT, AU, AO] or WA) according to age

| **Population and number of comorbidities** | | **AA, n (%)** | **[AT, AU, AO], or WA pooled, n (%)** |
| --- | --- | --- | --- |
| **Age** |  |  |  |
| 0–11 y | Patients with 1 comorbidity | 2025 (30.4) | 29 (24.4) |
|  | Patients with 2 or more comorbidities | 3234 (48.6) | 68 (57.1) |
| 12–17 y | Patients with 1 comorbidity | 1520 (36.2) | 34 (41.5) |
|  | Patients with 2 or more comorbidities | 1179 (28.1) | 26 (31.7) |
| 18–39 y | Patients with 1 comorbidity | 7578 (33.5) | 199 (33.1) |
|  | Patients with 2 or more comorbidities | 4946 (21.9) | 218 (36.2) |
| 40–59 y | Patients with 1 comorbidity | 7990 (32.3) | 218 (35.3) |
|  | Patients with 2 or more comorbidities | 7275 (29.4) | 235 (38.1) |
| ≥60 y | Patients with 1 comorbidity | 1083 (29.4) | 21 (27.3) |
|  | Patients with 2 or more comorbidities | 1503 (40.8) | 38 (49.4) |
| All years | Patients with 1 comorbidity | 20,196 (32.6) | 501 (33.5) |
|  | Patients with 2 or more comorbidities | 18,137 (29.3) | 585 (39.1) |

Abbreviations: AA, alopecia areata; AO, alopecia ophiasis; AT, alopecia totalis;
AU, alopecia universalis; WA, widespread alopecia; y, years

**TABLE S5** Treatment use 1 and 2 years after AA first diagnosis, stratified by age (N = 61,899)

|  | **Age group** | | | | |
| --- | --- | --- | --- | --- | --- |
| **Characteristic** | **0–11 y, n = 6657**  **(10.8% of patients)** | **12–17 y, n = 4199**  **(6.8% of patients)** | **18–39 y, n = 22,600**  **(36.5% of patients)** | **40–59 y, n = 24,757**  **(40% of patients)** | **≥60 y, n = 3686**  **(6% of patients)** |
| Male, n (%) | 2648 (39.8) | 1938 (46.2) | 10,436 (46.2) | 10,058 (40.6) | 1554 (42.2) |
| Female, n (%) | 4009 (60.2) | 2261 (53.8) | 12,164 (53.8) | 14,699 (59.4) | 2132 (57.8) |
| **Treatment use 1 year after diagnosis, %** |  |  |  |  |  |
| Corticosteroids (intralesional) | 0.3 | 2.8 | 5.8 | 6.3 | 5.0 |
| Corticosteroids (intravenous) | 0.0 | 0.3 | 0.4 | 0.4 | 0.2 |
| Pulse therapy (intravenous corticosteroids) | 0.0 | 0.3 | 0.4 | 0.4 | 0.1 |
| Corticosteroids (intramuscular) | 0.2 | 1.4 | 2.9 | 3.3 | 2.8 |
| Corticosteroids (oral) | 0.9 | 2.2 | 3.9 | 4.1 | 3.9 |
| Corticosteroids (topical) | 68.1 | 65.9 | 70.8 | 70.2 | 68.5 |
| Glycyrrhizin (oral) | 3.3 | 13.2 | 19.5 | 19.5 | 16.0 |
| Carpronium chloride (topical) | 34.9 | 38.7 | 36.5 | 37.6 | 38.1 |
| Cepharanthine (oral) | 11.8 | 29.9 | 39.1 | 41.2 | 36.7 |
| Antihistamine (oral) | 18.4 | 23.7 | 21.9 | 22.3 | 22.6 |
| Herbal medicine (oral) | 0.0 | 0.1 | 0.1 | 0.1 | 0.0 |
| **Treatment use 2 years after diagnosis, %** |  |  |  |  |  |
| Corticosteroids (intralesional) | 0.4 | 3.1 | 6.2 | 6.6 | 5.2 |
| Corticosteroids (intravenous) | 0.0 | 0.4 | 0.5 | 0.4 | 0.2 |
| Pulse therapy (intravenous corticosteroids) | 0.0 | 0.4 | 0.5 | 0.4 | 0.1 |
| Corticosteroids (intramuscular) | 0.2 | 1.6 | 3.2 | 3.6 | 2.9 |
| Corticosteroids (oral) | 0.9 | 2.5 | 4.2 | 4.4 | 4.2 |
| Corticosteroids (topical) | 68.9 | 67.0 | 71.7 | 71.2 | 69.7 |
| Glycyrrhizin (oral) | 3.3 | 13.5 | 20.0 | 19.9 | 16.2 |
| Carpronium chloride (topical) | 35.5 | 39.3 | 37.2 | 38.2 | 38.7 |
| Cepharanthine (oral) | 12.0 | 30.6 | 39.7 | 41.8 | 37.1 |
| Antihistamine (oral) | 19.4 | 24.6 | 22.8 | 23.6 | 23.7 |
| Herbal medicine (oral) | 0.0 | 0.1 | 0.1 | 0.1 | 0.0 |

Abbreviations: AA, alopecia areata; y, years.

**TABLE S6** Treatment use 1 and 2 years after ([AT, AU, AO] or WA) first diagnosis, stratified by age (N = 1497 patients)

|  | **Age group** | | | | | |
| --- | --- | --- | --- | --- | --- | --- |
| **Characteristic** | **0–11 y, n = 119**  **(8% of patients)** | | **12–17 y, n = 82**  **(5.5% of patients)** | **18–39 y, n = 602**  **(40.2% of patients)** | **40–59 y, n = 617**  **(41.2% of patients)** | **≥60 y, n = 77**  **(5.1% of patients)** |
| Male, n (%) | | 57 (47.9) | 38 (46.3) | 256 (42.5) | 244 (39.5) | 32 (41.6) |
| Female, n (%) | | 62 (52.1) | 44 (53.7) | 346 (57.5) | 373 (60.5) | 45 (58.4) |
| **Treatment use 1 year after diagnosis, %** | |  |  |  |  |  |
| Corticosteroids (intralesional) | | 0.8 | 4.9 | 12.0 | 10.7 | 7.8 |
| Corticosteroids (intravenous) | | 2.5 | 11.0 | 12.0 | 10.2 | 6.5 |
| Pulse therapy (intravenous corticosteroids) | | 2.5 | 11.0 | 11.8 | 9.9 | 5.2 |
| Corticosteroids (intramuscular) | | 0.0 | 1.2 | 4.8 | 5.2 | 1.3 |
| Corticosteroids (oral) | | 4.2 | 8.5 | 13.3 | 16.4 | 15.6 |
| Corticosteroids (topical) | | 69.7 | 63.4 | 61.8 | 64.2 | 66.2 |
| Glycyrrhizin (oral) | | 4.2 | 14.6 | 16.1 | 18.6 | 14.3 |
| Carpronium chloride (topical) | | 20.2 | 19.5 | 20.6 | 25.3 | 32.5 |
| Cepharanthine (oral) | | 10.1 | 30.5 | 35.5 | 38.4 | 36.4 |
| Antihistamine (oral) | | 26.1 | 35.4 | 29.2 | 32.1 | 27.3 |
| Herbal medicine (oral) | | 0.0 | 0.0 | 0.0 | 0.0 | 0.0 |
| **Treatment use 2 years after diagnosis, %** | |  |  |  |  |  |
| Corticosteroids (intralesional) | | 0.8 | 6.1 | 13.0 | 11.5 | 7.8 |
| Corticosteroids (intravenous) | | 2.5 | 11.0 | 12.5 | 10.7 | 6.5 |
| Pulse therapy (intravenous corticosteroids) | | 2.5 | 11.0 | 12.3 | 10.1 | 5.2 |
| Corticosteroids (intramuscular) | | 0.0 | 2.4 | 5.5 | 5.7 | 1.3 |
| Corticosteroids (oral) | | 5.0 | 9.8 | 14.6 | 17.0 | 15.6 |
| Corticosteroids (topical) | | 69.7 | 65.9 | 64.3 | 67.4 | 66.2 |
| Glycyrrhizin (oral) | | 4.2 | 15.9 | 16.6 | 19.0 | 15.6 |
| Carpronium chloride (topical) | | 20.2 | 22.0 | 21.3 | 25.8 | 32.5 |
| Cepharanthine (oral) | | 10.9 | 31.7 | 36.0 | 38.7 | 36.4 |
| Antihistamine (oral) | | 26.9 | 37.8 | 30.9 | 32.7 | 29.9 |
| Herbal medicine (oral) | | 0.0 | 0.0 | 0.0 | 0.0 | 0.0 |

Abbreviations: AO, alopecia ophiasis; AT, alopecia totalis; AU, alopecia universalis; WA, widespread alopecia.

**TABLE S7** Treatment use 1 and 2 years after AA first diagnosis, stratified by years (2012–2019); N = 61,899

|  | **Treatment use 1 and 2 years after AA first diagnosis** | | | | | | | |
| --- | --- | --- | --- | --- | --- | --- | --- | --- |
| **Characteristic** | **2012 n = 2879** | **2013  n = 4746** | **2014  n = 4370** | **2015  n = 7555** | **2016  n = 8329** | **2017 n = 10,021** | **2018  n = 12,293** | **2019  n = 11,706** |
| Male, n (%) | 1262 (43.8) | 2141 (45.1) | 1993 (45.6) | 3370 (44.6) | 3604 (43.3) | 4210 (42) | 5186 (42.2) | 4868 (41.6) |
| Female, n (%) | 1617 (56.17) | 2605 (54.9) | 2377 (54.4) | 4185 (55.4) | 4725 (56.7) | 5811 (58) | 7107 (57.8) | 6838 (58.4) |
| **Treatment use 1 year after diagnosis, %** |  |  |  |  |  |  |  |  |
| Corticosteroids (intralesional) | 4.4 | 4.6 | 4.1 | 5.0 | 5.1 | 5.3 | 5.8 | 5.2 |
| Corticosteroids (intravenous) | 0.0 | 0.2 | 0.3 | 0.3 | 0.3 | 0.4 | 0.3 | 0.6 |
| Pulse therapy (intravenous corticosteroids) | 0.0 | 0.2 | 0.3 | 0.3 | 0.3 | 0.4 | 0.3 | 0.5 |
| Corticosteroids (intramuscular) | 3.3 | 3.1 | 2.5 | 3.1 | 2.7 | 2.8 | 2.7 | 2.0 |
| Corticosteroids (oral) | 3.5 | 4.1 | 3.0 | 4.0 | 4.0 | 3.7 | 3.6 | 2.7 |
| Corticosteroids (topical) | 64.6 | 66.8 | 68.1 | 68.1 | 68.7 | 71.1 | 72.2 | 71.1 |
| Glycyrrhizin (oral) | 17.5 | 17.5 | 17.7 | 17.2 | 17.2 | 17.6 | 16.8 | 16.4 |
| Carpronium chloride (topical) | 41.3 | 39.1 | 41.6 | 37.1 | 37.6 | 37.6 | 35.1 | 34.4 |
| Cepharanthine (oral) | 38.7 | 37.0 | 38.2 | 36.6 | 36.5 | 36.3 | 35.9 | 34.6 |
| Antihistamine (oral) | 21.7 | 21.8 | 20.3 | 22.9 | 22.2 | 22.4 | 22.5 | 20.2 |
| Herbal medicine (oral) | 0.0 | 0.0 | 0.1 | 0.1 | 0.1 | 0.1 | 0.1 | 0.1 |
| **Treatment use 2 years after diagnosis, %** |  |  |  |  |  |  |  |  |
| Corticosteroids (intralesional) | 4.8 | 5.1 | 4.4 | 5.4 | 5.5 | 5.8 | 6.0 | 5.2 |
| Corticosteroids (intravenous) | 0.0 | 0.2 | 0.3 | 0.4 | 0.3 | 0.5 | 0.3 | 0.6 |
| Pulse therapy (intravenous corticosteroids) | 0.0 | 0.2 | 0.3 | 0.4 | 0.3 | 0.5 | 0.3 | 0.5 |
| Corticosteroids (intramuscular) | 3.7 | 3.4 | 2.8 | 3.4 | 2.9 | 3.1 | 2.8 | 2.0 |
| Corticosteroids (oral) | 3.8 | 4.9 | 3.3 | 4.3 | 4.3 | 4.0 | 3.8 | 2.7 |
| Corticosteroids (topical) | 66.2 | 68.4 | 69.3 | 69.4 | 70.1 | 72.5 | 72.9 | 71.1 |
| Glycyrrhizin (oral) | 18.2 | 18.1 | 18.3 | 17.7 | 17.7 | 18.2 | 17.1 | 16.4 |
| Carpronium chloride (topical) | 42.2 | 40.0 | 42.4 | 38.0 | 38.5 | 38.6 | 35.5 | 34.4 |
| Cepharanthine (oral) | 39.7 | 38.0 | 39.2 | 37.2 | 37.2 | 37.0 | 36.2 | 34.6 |
| Antihistamine (oral) | 23.1 | 23.6 | 21.5 | 24.5 | 23.8 | 23.9 | 23.4 | 20.2 |
| Herbal medicine (oral) | 0.0 | 0.0 | 0.1 | 0.1 | 0.1 | 0.1 | 0.1 | 0.1 |

Abbreviation: AA, alopecia areata.

**TABLE S8** Treatment use 1 and 2 years after ([AT, AU, AO] or WA) first diagnosis, stratified by years (2012–2019); N = 1497

|  | **Treatment Use 1 and 2 Years After ([AT, AU, AO] or WA) First Diagnosis** | | | | | | | |
| --- | --- | --- | --- | --- | --- | --- | --- | --- |
| **Characteristic** | **2012 n = 46** | **2013 n = 103** | **2014 n = 88** | **2015 n = 192** | **2016 n = 176** | **2017 n = 252** | **2018 n = 330** | **2019 n = 310** |
| Male, n (%) | 17 (40) | 38 (36.9) | 35 (39.8) | 93 (48.4) | 77 (43.8) | 102 (40.5) | 136 (41.2) | 129 (41.6) |
| Female, n (%) | 29 (60) | 65 (63.1) | 53 (60.2) | 99 (51.6) | 99 (56.2) | 150 (59.5) | 194 (58.8) | 181 (58.4) |
| **Treatment use 1 year after diagnosis, %** |  |  |  |  |  |  |  |  |
| Corticosteroids (intralesional) | 4.3 | 8.7 | 4.5 | 6.8 | 13.6 | 11.1 | 11.5 | 10.0 |
| Corticosteroids (intravenous) | 2.2 | 3.9 | 9.1 | 8.3 | 6.8 | 9.5 | 8.8 | 18.7 |
| Pulse therapy (intravenous corticosteroids) | 2.2 | 3.9 | 9.1 | 8.3 | 5.7 | 9.5 | 8.8 | 18.1 |
| Corticosteroids (intramuscular) | 6.5 | 3.9 | 3.4 | 5.2 | 6.2 | 4.4 | 3.3 | 3.2 |
| Corticosteroids (oral) | 17.4 | 15.5 | 17.0 | 11.5 | 16.5 | 14.7 | 12.4 | 11.9 |
| Corticosteroids (topical) | 60.9 | 63.1 | 63.6 | 55.2 | 65.3 | 64.7 | 65.5 | 66.1 |
| Glycyrrhizin (oral) | 19.6 | 21.4 | 20.5 | 16.7 | 14.2 | 12.3 | 17.3 | 14.8 |
| Carpronium chloride (topical) | 41.3 | 33.0 | 34.1 | 20.3 | 24.4 | 20.6 | 19.4 | 20.6 |
| Cepharanthine (oral) | 45.7 | 40.8 | 46.6 | 35.9 | 34.7 | 31.7 | 33.3 | 29.7 |
| Antihistamine (oral) | 32.6 | 27.2 | 29.5 | 29.2 | 36.9 | 33.7 | 29.7 | 26.5 |
| Herbal medicine (oral) | 0.0 | 0.0 | 0.0 | 0.0 | 0.0 | 0.0 | 0.0 | 0.0 |
| **Treatment use 2 years after diagnosis, %** |  |  |  |  |  |  |  |  |
| Corticosteroids (intralesional) | 4.3 | 10.7 | 4.5 | 8.3 | 14.8 | 12.3 | 12.1 | 10.0 |
| Corticosteroids (intravenous) | 2.2 | 3.9 | 9.1 | 9.4 | 7.4 | 9.9 | 9.4 | 18.7 |
| Pulse therapy (intravenous corticosteroids) | 2.2 | 3.9 | 9.1 | 9.4 | 5.7 | 9.9 | 9.1 | 18.1 |
| Corticosteroids (intramuscular) | 6.5 | 4.9 | 3.4 | 6.8 | 6.8 | 5.2 | 3.6 | 3.2 |
| Corticosteroids (oral) | 17.4 | 16.5 | 17.0 | 14.1 | 17.0 | 16.7 | 13.0 | 11.9 |
| Corticosteroids (topical) | 63.0 | 64.1 | 64.8 | 62.5 | 69.3 | 68.3 | 66.7 | 66.1 |
| Glycyrrhizin (oral) | 19.6 | 23.3 | 20.5 | 18.2 | 14.8 | 12.3 | 17.6 | 14.8 |
| Carpronium chloride (topical) | 41.3 | 33.0 | 34.1 | 22.4 | 26.1 | 20.6 | 20.0 | 20.6 |
| Cepharanthine (oral) | 45.7 | 42.7 | 47.7 | 37.0 | 35.2 | 31.7 | 33.6 | 29.7 |
| Antihistamine (oral) | 32.6 | 28.2 | 31.8 | 32.3 | 40.3 | 34.5 | 30.3 | 26.5 |
| Herbal medicine (oral) | 0.0 | 0.0 | 0.0 | 0.0 | 0.0 | 0.0 | 0.0 | 0.0 |

Abbreviations: AO, alopecia ophiasis; AT, alopecia totalis; AU, alopecia universalis; WA, widespread alopecia.

**TABLE S9** Patients with one-time prescription only

|  | **AA** | | **[AT, AU, AO] or WA Pooled** | | |
| --- | --- | --- | --- | --- | --- |
| **Medicine** | **Number of patients** | **One-time prescription only  n (%)** | | **Number of patients** | **One-time prescription only**  **n (%)** |
| Corticosteroids (intralesional) | 3379 | 900 (26.6) | | 161 | 29 (18) |
| Pulse therapy (intravenous corticosteroids) | 220 | 188 (85.5) | | 152 | 127 (83.6) |
| Corticosteroids (intramuscular) | 1778 | 526 (29.6) | | 71 | 13 (18.3) |
| Corticosteroids (oral) | 2351 | 538 (22.9) | | 219 | 47 (21.5) |
| Corticosteroids (topical) | 43,803 | 15,055 (34.4) | | 991 | 279 (28.2) |
| Glycyrrhizin (oral) | 10,832 | 3176 (29.3) | | 247 | 50 (20.2) |
| Carpronium chloride (topical) | 23,315 | 12,099 (51.9) | | 354 | 137 (38.7) |
| Cepharanthine (oral) | 22,781 | 6807 (29.9) | | 523 | 110 (21) |
| Antihistamine (oral) | 14,193 | 4823 (34) | | 474 | 119 (25.1) |
| Herbal medicine (oral) | 49 | 16 (32.7) | | 0 | 0 |

Abbreviations: AA, alopecia areata; AO, alopecia ophiasis; AT, alopecia totalis; AU, alopecia universalis; WA, widespread alopecia.


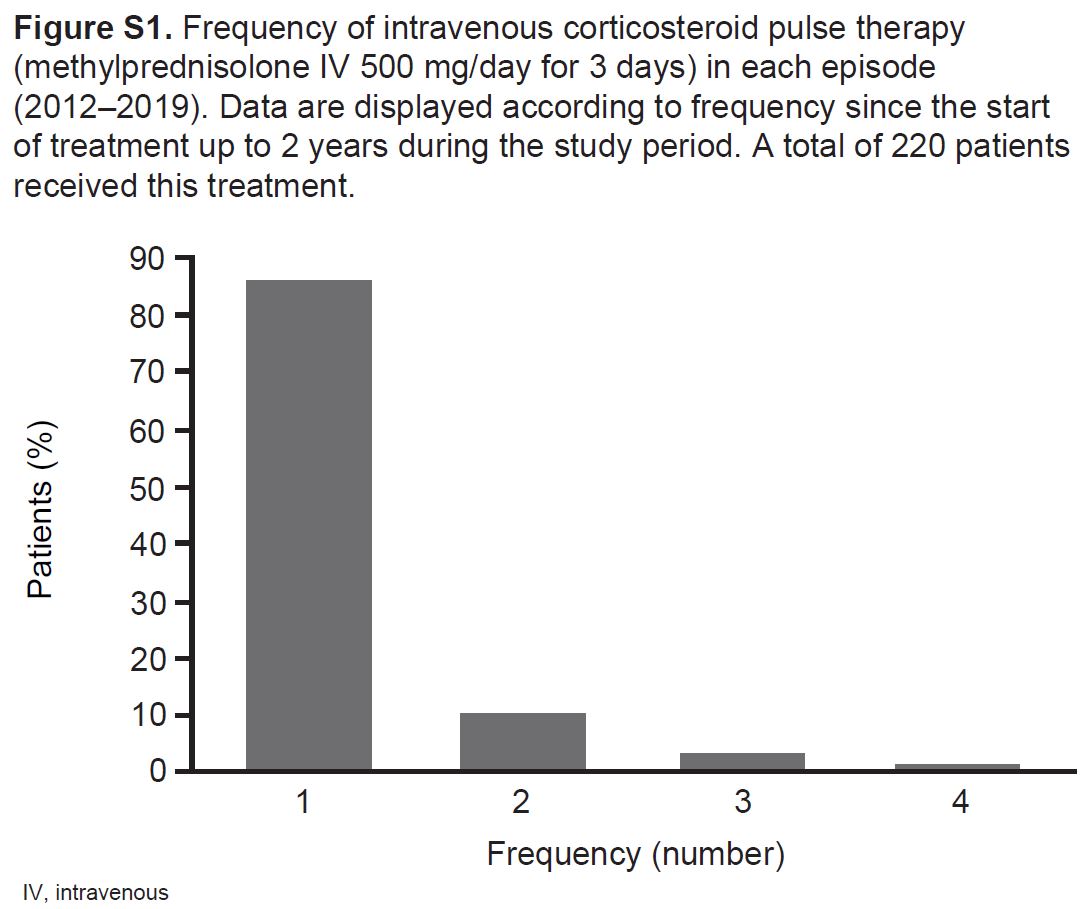

Supplement: Supplementary file 1 — Tables S1‐S9 [file JDE-50-37-s001.docx]
